# Lifespan and healthspan benefits of exogenous H_2_S in *C. elegans* are independent from effects downstream of *eat-2* mutation

**DOI:** 10.1038/s41514-020-0044-8

**Published:** 2020-06-10

**Authors:** Li Theng Ng, Li Fang Ng, Richard Ming Yi Tang, Diogo Barardo, Barry Halliwell, Philip Keith Moore, Jan Gruber

**Affiliations:** 1 Ageing Research Laboratory, Science Division, Yale-NUS College, Singapore, 138527 Singapore; 2 Department of Pharmacology, Yong Loo Lin School of Medicine, National University of Singapore, Singapore, 117600 Singapore; 3 Department of Biochemistry, Yong Loo Lin School of Medicine, National University of Singapore, Singapore, 117596 Singapore; 4 Neurobiology Programme, Life Sciences Institute, National University of Singapore, Singapore, 117456 Singapore; 5 NUS Graduate School for Integrative Sciences & Engineering, National University of Singapore, Singapore, 117456 Singapore

**Keywords:** Ageing, Drug discovery, Ageing

## Abstract

Caloric restriction (CR) is one of the most effective interventions to prolong lifespan and promote health. Recently, it has been suggested that hydrogen sulfide (H_2_S) may play a pivotal role in mediating some of these CR-associated benefits. While toxic at high concentrations, H_2_S at lower concentrations can be biologically advantageous. H_2_S levels can be artificially elevated *via* H_2_S-releasing donor drugs. In this study, we explored the function of a novel, slow-releasing H_2_S donor drug (FW1256) and used it as a tool to investigate H_2_S in the context of CR and as a potential CR mimetic. We show that exposure to FW1256 extends lifespan and promotes health in *Caenorhabditis elegans* (*C. elegans*) more robustly than some previous H_2_S-releasing compounds, including GYY4137. We looked at the extent to which FW1256 reproduces CR-associated physiological effects in normal-feeding *C. elegans*. We found that FW1256 promoted healthy longevity to a similar degree as CR but with fewer fitness costs. In contrast to CR, FW1256 actually enhanced overall reproductive capacity and did not reduce adult body length. FW1256 further extended the lifespan of already long-lived *eat-2* mutants without further detriments in developmental timing or fertility, but these lifespan and healthspan benefits required H_2_S exposure to begin early in development. Taken together, these observations suggest that FW1256 delivers exogenous H_2_S efficiently and supports a role for H_2_S in mediating longevity benefits of CR. Delivery of H_2_S *via* FW1256, however, does not mimic CR perfectly, suggesting that the role of H_2_S in CR-associated longevity is likely more complex than previously described.

## Introduction

The fraction of aged individuals in many populations around the world is increasing more rapidly than at any time in human history^1^. As a result of this unprecedented demographic change, there is a growing need to identify efficacious, low-risk interventions that promote healthy longevity. A major challenge in biomedical research today is to develop safe, efficacious therapies that can extend human healthspan by delaying or preventing ageing-associated diseases.

Caloric restriction (CR), restricting the amount of calories consumed without causing malnutrition, is one of only a few interventions that have been shown to extend lifespan and healthspan in a wide range of model organisms, ranging from unicellular yeast^2^ to multicellular organisms including flies^3^, nematodes^4^ and rodents^5^. While there are no direct data on longevity effects of CR in humans, findings from rhesus monkeys imply that CR likely also benefits lifespan and certainly healthspan in primates^6–8^. Evidence in humans also supports such health benefits, for example, CALERIE (Comprehensive Assessment of the Long-term Effects of Reducing Intake of Energy), a two year study of caloric restriction in humans conducted by the National Institute of Aging (NIA), has confirmed that CR is both feasible in humans and benefits a number of health indicators^9^. However, restricting food intake in humans is notoriously difficult and fraught with physiological and psychological challenges^10^. CR diets are extremely difficult to maintain over long periods of time and outside of carefully controlled experiments, there is a significant risk of inadvertently causing malnutrition^11^. This is of particular concern during human ageing, where malnutrition is already known to be a common problem^12^. Encouraging CR as a means of reducing morbidity in ageing populations is therefore probably not a viable strategy. However, a recent study has shown that extended daily fasting periods can benefit lifespan, regardless of dietary composition or total calories consumed, opening up potential alternative approaches to improve healthspan^13^. Insights into the mechanisms by which CR delays or prevents ageing-associated diseases and extends lifespan may provide another alternative strategy. One promising approach to extending healthspan would be to identify compounds that reproduce CR-associated benefits without the need of adhering to an actual CR regime^14–16^. In this respect, hydrogen sulfide (H_2_S) has recently been reported to act as a potential mediator of CR-associated benefits^17^.

H_2_S is a poisonous, water-soluble gas with a pungent odor of rotten eggs. At levels above 50ppm in air, H_2_S is acutely toxic to humans, whilst levels in excess of 300ppm are potentially fatal^18^. However, it is now clear that H_2_S is produced endogenously and that low endogenous levels may play a role in many biological processes^19,20^. H_2_S is synthesized endogenously in mammalian tissues by three types of enzymes: cystathionine-γ-lyase (CSE), cystathionine β-synthase (CBS) and 3-mercaptopyruvate sulfurtransferase (3-MST). Importantly, H_2_S has been shown to be involved in several physiological and pathophysiological processes closely associated with ageing, including inflammation^21,22^, cancer^23^ and atherosclerosis^24^.

Interestingly, recent evidence in *Caenorhabditis elegans* (*C. elegans*), yeast, flies and mice suggest that H_2_S may also play an evolutionarily conserved role in lifespan determination and in mediating CR benefits. Miller and Roth first showed that exposure to H_2_S gas significantly extends lifespan and improves thermo-tolerance in *C. elegans*^25^. Later, Hine et al*.* reported that one of the *C. elegans* orthologues of the mammalian CBS enzyme, *cbs-1*, is required for the extended lifespan of *C. elegans eat-2* mutants^17^. CR also significantly induces endogenous H_2_S production in fruit flies, nematodes, yeast and mice^17^. In addition, *cbs-1* has been reported to be required for the long-lifespan of germline-deficient *glp-1* nematodes^26^. Reducing *cbs-1* expression in *glp-1* mutants was found to decrease H_2_S levels and shorten their lifespan^26^. H_2_S has therefore been implicated in lifespan modulation not only in CR, but also in response to germline signaling. However, whether either mechanism is related to the lifespan effects following lifelong exposure to exogenous H_2_S remains to be shown. In mice, CR has been shown to transcriptionally induce the CSE enzyme and this has been suggested to be a mechanism underlying protection against ischemia-reperfusion injury, as evidenced by a failure in protection against ischemia-reperfusion injury following CR in CSE knockout mice^17^. It therefore seems that increased H_2_S synthesis may be an evolutionarily conserved mechanism, involved in mediating physiological benefits under CR conditions^17^. However, whether H_2_S acts as a signaling molecule or plays a more direct role in modulating CR-associated benefits remains to be determined. Nevertheless, there is increasing evidence that enhancing endogenous H_2_S levels can be physiologically beneficial. A particularly intriguing idea is that H_2_S donor drugs, or modulators of pathways involved in H_2_S metabolism, might represent avenues for the development of CR mimetics^27^.

Several inorganic compounds can be utilized to generate H_2_S exogenously. For example sodium hydrosulfide (NaHS) releases H_2_S rapidly upon hydrolysis and can be used to deliver H_2_S in vivo^28^. However, during CR, physiological exposure to H_2_S is likely at low-levels and chronic, unlike exposure resulting from the rapid release of H_2_S from NaHS. Several novel synthetic organic H_2_S donors have therefore been developed. Such compounds are designed to release H_2_S slowly over an extended period of time; an approach which likely better approximates endogenous H_2_S release in vivo. One of the first was GYY4137 (morpholin-4-ium-4-methoxyphenyl [morpholino] phosphinodithioate), a compound that releases H_2_S slowly both in vitro and in vivo^29^. Since it was first described, GYY4137 has been studied extensively and shown to exert biological effects in mice, rats and nematodes^29–33^. In recent years, AP39, a H_2_S donor designed to release H_2_S specifically within mitochondria has been developed^34^ and shown to confer cytoprotective activity and to attenuate the loss of cellular bioenergetics in cells subjected to oxidative stress^35^. AP39 has been reported to elicit protection effects against renal ischemia-reperfusion injury in rats^36^ and preserve mitochondrial function in APP/PS1 mice and neurons, indicating a potential role in protecting against Alzheimer’s disease^37^.

We have developed a series of novel H_2_S donors. Among these H_2_S donors, 3-dihydro-2-phenyl-sulfanylenebenzo[d] [1,3,2]-oxazaphosphole (FW1256) exerts superior anti-proliferative effects compared to GYY4137, as evaluated in MCF7 breast cancer cells^38^. FW1256 has also been shown to exert significant anti-inflammatory effects in RAW264.7 macrophages^39^. However, the efficacy of these novel H_2_S donors in the context of ageing remains to be explored.

Here we report the effects of two novel H_2_S donor compounds (FW1251 and FW1256) on lifespan, healthspan and other CR-associated phenotypes in nematodes. We observed that FW1256 extended lifespan and promoted healthy ageing in wild-type (WT) *C. elegans* but did not fully replicate CR-associated effects. We found that, in *C. elegans*, exogenous H_2_S promoted healthy longevity with less severe detrimental effects on some parameters of fitness than CR as modelled by *eat-2* mutation. FW1256 was also able to further extend the already long lifespan of *eat-2* mutants. Finally, we provide evidence that H_2_S affects mitochondrial function and that exposure to H_2_S during larval development was required for these benefits, suggesting involvement of a developmental adaptation. Our data imply that the function of H_2_S in lifespan regulation and CR is more complex than previously appreciated.

## Results

### Screen for novel H_2_S donor compounds with lifespan extension effect

It was previously reported that GYY4137 (morpholin-4-ium-4-methoxyphenyl [morpholino] phosphinodithioate), a slow-releasing H_2_S donor, extends lifespan in *C. elegans*^33^ but the effect size, even after careful dose optimization, was small compared to than that previously reported by Miller and Roth for direct exposure to H_2_S gas^25^. We wondered if a H_2_S donor compound with a more rapid H_2_S release rate might elicit effects more comparable to those achieved by Miller and Roth. For the current study, we therefore selected two compounds from a series of our novel H_2_S donor compounds, FW1251 and FW1256 (Table 1). Both FW1251 and FW1256 release H_2_S more rapidly than GYY4137^38^. Both compounds were designed primarily to deliver exogenous H_2_S to maximize anti-proliferative activity against cancer cells^38^. While GYY4137 releases H_2_S at a rate of about 1% per day under standard assay conditions (acetonitrile [MeCN]:phosphate buffered saline [PBS] at room temperature), FW1251 releases H_2_S twice as fast under the same condition and FW1256 releases H_2_S even more rapidly, releasing about 5% per day^38^. The mechanism for H_2_S release by FW1256 in aqueous environment is shown in schematic form in Supplementary Fig. 1. Release of H_2_S from another H_2_S donor, diallyl trisulfide (DATS), has been shown to be highly dependent on the presence of biological thiols^40^. It is therefore, worth noting that biological thiols might affect the efficacy and actual release rate of H_2_S from donor compounds in vivo. However, previous data suggest that FW1256 is more potent as an anticancer agent than GYY4137 yet is nontoxic to human lung fibroblast cells (WI38) even at 500μM^38^.

**Table 1:**
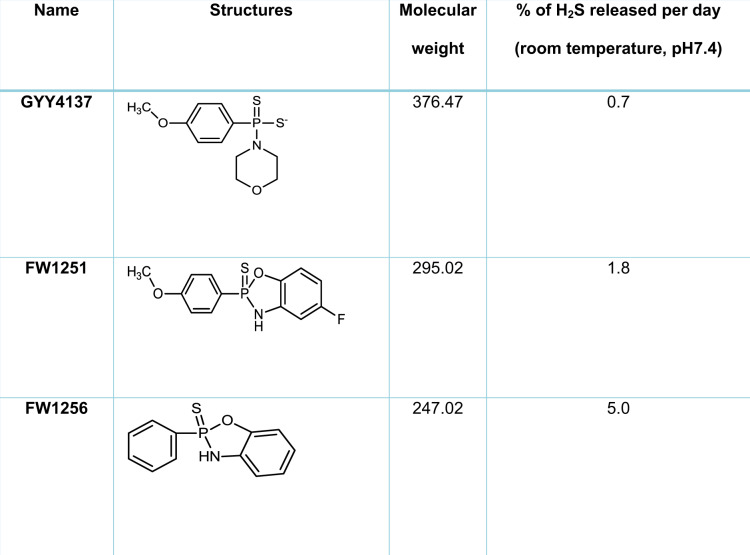
Chemical structures and H_2_S releasing speeds of H_2_S donor compounds used in this study compared to GYY4137.

**Fig. 1:**
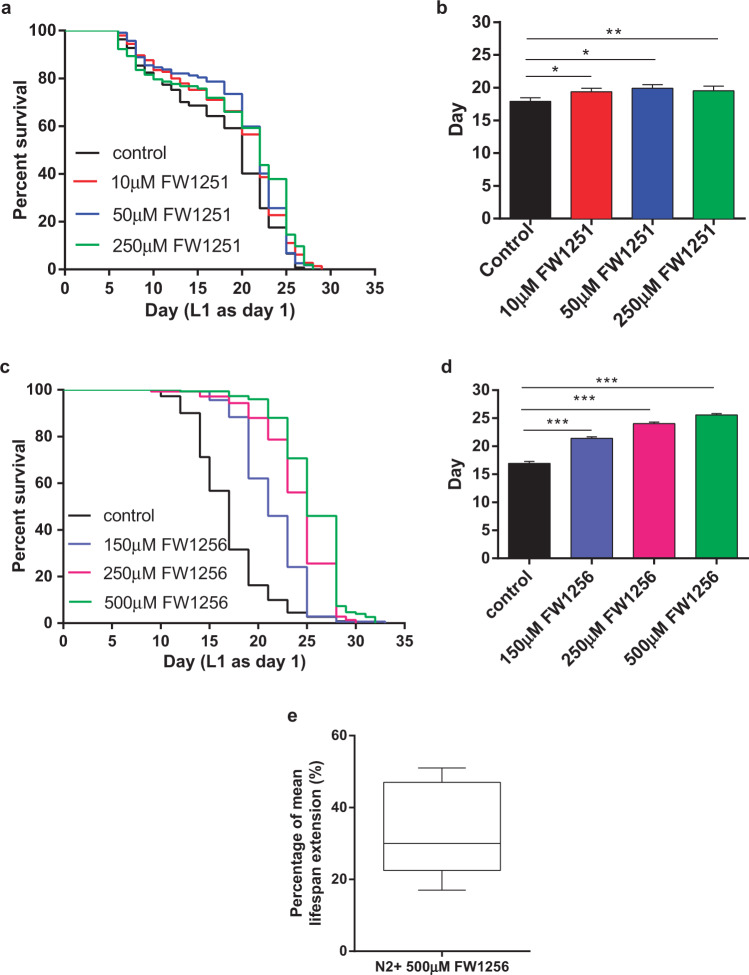
Effects of FW1251 and FW1256 on lifespan of WT *C. elegans*. **a** Survival curves and **b** mean lifespan of WT *C. elegans* exposed to different doses of FW1251, *n* = 103–145 animals per condition. **c**, **d** Dose–response experiment of FW1256 on lifespan of WT *C. elegans*, **c** survival curves, **d** mean lifespan, *n* = 111–150 animals per condition. **e** Boxplot showing distribution of mean lifespan extension effects on WT *C. elegans* upon exposure to 500 µM of FW1256 from the five independent experiments (Supplementary Table 1). In this box plot, the bottom and top of the box represent the 25% percentile and 75% percentile, the center line represents the median and the whiskers encompass the entire range of values for all five independent experiments. (Survival curves were analyzed using log-rank tests while mean lifespans were analyzed using OASIS2, *p < 0.05, **p < 0.01, ***p < 0.001).

To explore effects of these compounds in *C. elegans*, we first optimized dose-response in terms of lifespan in *C. elegans*. We carried out range finding for optimal dose for both compounds. WT *C. elegans* were exposed to four different concentrations (10 µM, 50 µM, 250 µM and 750 µM) of FW1251 in a series of operator-blinded lifespan studies. We observed no obvious toxicity below 750 µM and found that 50 µM resulted in the largest beneficial effect within this range (log-rank test, *p* < 0.05) (Fig. 1a). However, mean lifespan was increased by only 11% even at the ideal dose (mean lifespan: 19.9 ± 0.5 days, compared to control: 17.9 ± 0.5 days, *p* < 0.05 (Fig. 1b, Supplementary Table 2). At 750 µM, FW1251 showed some toxic effect on WT *C. elegans* (Supplementary Fig. 2, Supplementary Table 2). FW1251 therefore provided only limited benefits at low concentrations and was ineffective or toxic at high doses (Supplementary Fig. 3).

For our second (and faster-releasing) compound, FW1256, *C. elegans* were exposed to 10 µM, 50 µM and 150 µM during the initial screening. The highest concentration, 150 µM, significantly extended lifespan while the lower concentrations were ineffective (Supplementary Fig. 4, Supplementary Table 2). Thereafter, *C. elegans* were exposed to even higher concentrations of FW1256 (250 µM and 500 µM) to establish an optimal dose and test for possible toxicity at high levels of FW1256. Surprisingly, FW1256 was beneficial at both of these concentrations (Fig. 1c). FW1256 (250 μM) resulted in 42% mean lifespan extension (mean lifespan: 24.0 ± 0.3 days, compared to control: 16.9 ± 0.4 days, *p* < 0.001) while FW1256 (500 µM) extended lifespan by 51% (mean lifespan: 25.6 ± 0.3 days, compared to control: 16.9 ± 0.4 days, *p* < 0.001) (Fig. 1d, Supplementary Table 2). While 500 µM of FW1256 resulted in the largest overall benefit, doubling concentration from 250 µM to 500 µM of FW1256 resulted in an additional increase of only 9% of mean lifespan. For the remainder of this study, we therefore focused on FW1256 at a dose of 500 µM (Supplementary Fig. 3).

Comparison with over 400 compounds from an exhaustive database of drugs with published evidence for effects on lifespan (DrugAge Build3) puts FW1256 into the top 15 of all 400 drugs (top 4%) in terms of efficacy in *C. elegans*^41^. However, lifespan effects in *C. elegans* can be highly variable^41^. In order to accurately quantify the lifespan extension effect of FW1256, a total of five independent (biologically independent cohorts of WT *C. elegans*), operator-blinded repeats of the lifespan assay at 500 µM were carried out. While lifespan effect size showed variability between repeats, FW1256 consistently extended mean lifespan in all 5 of these independent experiments (mean effect size: 33.8%; 95% confidence interval: 17.3%–50.4%, Supplementary Table 1, Fig. 1e).

### FW1256 releases H_2_S in vivo

**Fig. 2:**
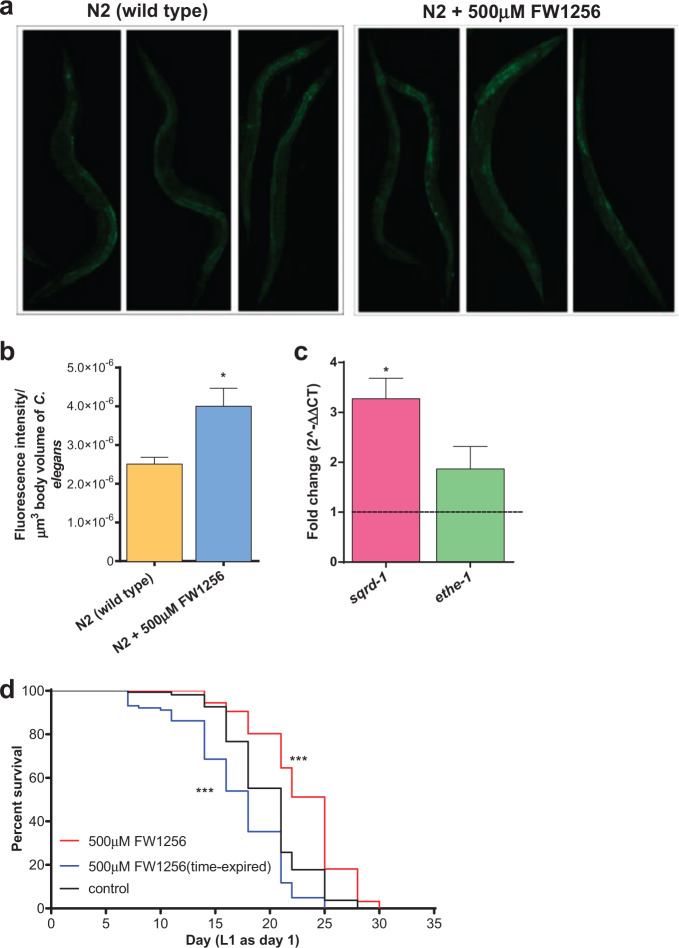
H_2_S moiety from FW1256 is the key of longevity increase. **a** Confocal microscopy fluorescence images of H_2_S detection using AzMC (in green) in *C. elegans* **b** Fluorescence intensity of the AzMC signal, normalized to body volume of *C. elegans* (*n* = 5 animals per condition, *t*-test, *p < 0.05)**. c** Relative quantification mRNA levels of *sqrd-1* and *ethe-1* encoding for endogenous H_2_S detoxification system in WT *C. elegans* exposed to 500 µM FW1256. Data show expression of *sqrd-1* and *ethe-1* relative to WT control (*n* = 3 repeated experiments, one sample *t*-test, *p < 0.05). **d** Effect of time-expired FW1256 (500 µM, 4 weeks at room temperature) on lifespan of WT *C. elegans* (*n* = 102–163 animals per condition, log-rank tests, ***p < 0.001).

Having established that FW1256 extends lifespan of *C. elegans*, we wondered if this effect was indeed due to the release of H_2_S in *C. elegans*. To test whether FW1256 was able to deliver H_2_S in vivo in *C. elegans*, we first used a fluorescence H_2_S sensor probe, 7-azido-4-methylcoumarin (AzMC)^42^. Fluorescence intensity of AzMC was significantly increased in WT *C. elegans* exposed to 500 µM FW1256 (Fig. 2a, b), confirming successful delivery of detectable quantities of exogenous H_2_S by FW1256. The amount of increase in H_2_S-related fluorescence seen in *eat-2* mutants is comparable to the FW1256-induced increase as judged by AzMC in WT *C. elegans* exposed to 500 µM of FW1256 (Supplementary Fig. 5). This comparison suggested that the amount of endogenous H_2_S resulting in nematodes exposed to 500 µM of FW1256 was approximately half of the increase seen in *eat-2* mutants (Supplementary Fig. 5).

We next monitored gene expression changes of two H_2_S oxidation enzymes, SQRD-1 and ETHE-1. SQRD-1 is an oxidoreductase that oxidizes H_2_S to polysulfide, which is then further oxidized to sulfate and thiosulfate *via* catalysis by ETHE-1^43^. Both of these enzymes are therefore directly involved in detoxifying/degrading H_2_S, contributing to maintaining intrinsic H_2_S levels^43^. While *sqrd-1* has previously been shown to be transcriptionally upregulated in response to the presence of H_2_S gas, *ethe-1* was not induced significantly under these conditions^43^. We nevertheless tested for potential upregulation of both *sqrd-1* and *ethe-1* mRNA upon exposure to 500 µM of FW1256. Consistent with delivery of physiologically relevant levels of H_2_S gas, mRNA levels of *sqrd-1* were increased by about 3 fold (*p* < 0.05) while mRNA levels of *ethe-1* were not upregulated significantly (about 2 fold change, *p* = 0.1952) in WT *C. elegans* exposed to 500 µM of FW1256 (Fig. 2c). Together, these data supported the notion that FW1256 delivers physiologically significant levels of H_2_S to *C. elegans*.

### The H_2_S moiety is required for FW1256 lifespan benefits

Next, we tested whether the lifespan enhancing effect of FW1256 was dependent on the presence of the H_2_S moiety in the molecule. To rule out possible lifespan benefits related to pharmacological effects of FW1256 independent of H_2_S, we carried out a control lifespan study with time-expired FW1256. Prior to exposing WT *C. elegans* to FW1256, FW1256 was allowed to expire for 4 weeks at room temperature in dimethyl sulfoxide (DMSO) to ensure that H_2_S was quantitatively released^44^ (For more information on this approach, see Supplementary Fig. 6). We found that time-expired FW1256 did not extend the lifespan of *C. elegans*. Time-expired FW1256, in fact, shortened lifespan (log-rank test, *p* < 0.001, mean ± SEM: 17.1 ± 0.4 days, compared to control: 19.9 ± 0.3 days, *p* < 0.001) (Fig. 2d, Supplementary Table 2), suggesting some toxicity of the expired compound in *C. elegans*. Together these data imply that fresh FW1256 delivers significant amounts of H_2_S exogenously to *C*. *elegans* and that the ability to release H_2_S is required for its lifespan benefits.

### FW1256 rescues detrimental effects and extends lifespans of H_2_S mutants

*C. elegans* possesses three families of endogenous H_2_S synthesizing enzymes, comprising two orthologues, each, of the mammalian CBS enzyme (*cbs-1*, *cbs-2*) and CSE enzyme (*cth-1*, *cth-2*) as well as seven orthologues of 3-MST (*mpst-1-7*)^33^. Both CBS and CSE require L-cysteine as substrate for H_2_S production and act mainly in cytoplasm while 3-MST converts 3-mercaptopyruvate to H_2_S, predominantly in the mitochondria (Supplementary Fig. 7)^45,46^.

**Fig. 3:**
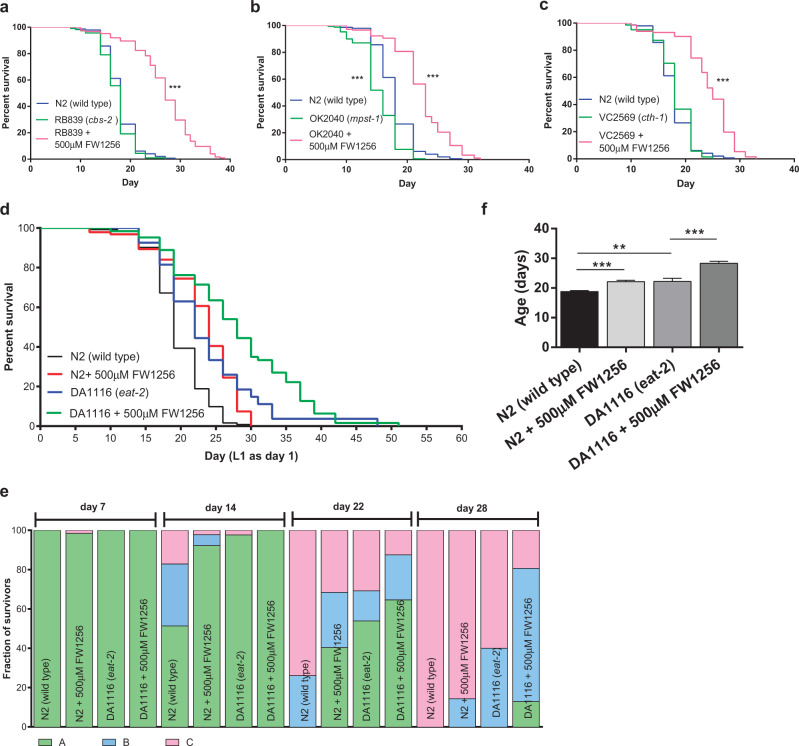
FW1256 not only benefits lifespans of H_2_S mutants and *eat-2* mutants, but also promotes health. **a–c** Comparison of lifespans in WT *C. elegans*, H_2_S mutants and H_2_S mutants exposed to FW1256 (500 µM) (data show log-rank tests, ***p < 0.001). **a** RB839, *cbs-2* mutants, *n* = 115-147 animals, **b** OK2040, *mpst-1* mutants, *n* = 147–181 animals, **c** VC2569, *cth-1* mutants, *n* = 132–147 animals. **d** Effect of FW1256 (500 µM) on lifespans of WT and *eat-2* mutants, *n* = 27–122 animals per condition. **e** Mobility status of surviving nematodes at different ages, *n* = 27–122 animals per condition. Generally, class A nematodes moved constantly, class B nematodes moved only when prodded while class C nematodes showed movement of their head and tails only. **f** Mean healthspan of unexposed and FW1256-exposed WT and *eat-2* mutants (*n* = 27–122 animals per condition, one-way ANOVA with Bonferroni’s post-test, **p < 0.01, ***p < 0.001).

We have previously shown that deletion of *mpst-1* shortens the lifespan of *C. elegans*^33^, suggesting a role of H_2_S production in the mitochondria in the context of normal lifespan determination. Whilst there is no prior report regarding the effect of *cbs-2* deletion on lifespan, *C. elegans* with *cbs-2* deletion has been reported to develop normally^47^. By contrast, RNA interference (RNAi) knockdown of *cth-1* has been reported to be detrimental to the lifespan of WT *C. elegans*^17^. These data suggest that H_2_S production in cytoplasm may also be required for normal lifespan. To quantitatively compare the importance of H_2_S production in different cellular compartments for lifespan regulation, we systematically compared the lifespan of three different H_2_S mutants: RB839 (*cbs-2*), OK2040 (*mpst-1*) and VC2569 (*cth-1*) under the standard conditions used in our laboratory. In our hands, the lifespans of *cbs-2* and *cth-1* mutants were unchanged, while a statistically significant shortened lifespan was observed consistently only in *mpst-1* mutants (log-rank test, *p* < 0.001, mean ± SEM: 15.4 ± 0.2 days, compared to WT: 17.9 ± 0.3 days, *p* < 0.001) (Fig. 3a–c, Supplementary Table 2), confirming that endogenous H_2_S production, at least in mitochondria, is required for normal lifespan in *C. elegans*.

To investigate if FW1256 rescues or extends the lifespan of these H_2_S mutants, three H_2_S mutant strains were exposed to FW1256 (500 µM). Interestingly, exposure to FW1256 extended lifespan in these mutants beyond that even of untreated WT animals (Fig. 3a–c). This further supports the notion that FW1256 effectively delivers H_2_S in vivo. Interestingly, comparison of FW1256 benefits between the three H_2_S mutant strains showed that lifespan benefits were most pronounced in the *cbs-2* mutant strain (log rank test, *p* < 0.001 with mean lifespan extension of 56%, mean ± SEM: 26.9 ± 0.5 days, compared to RB839 control: 17.2 ± 0.3 days, *p* < 0.001) (Fig. 3a, Supplementary Table 2), perhaps suggesting that loss of cytosolic *cbs* leads to compensatory effects that prime animals for lifespan extension by exogenously delivered H_2_S.

### Similar to CR, FW1256 promotes healthy longevity in *C. elegans*

In *C. elegans*, CR can be modelled genetically by mutations that reduce pharyngeal pumping rate, thereby decreasing the amount of food consumed^4^. It has previously been reported that one *C. elegans* endogenous H_2_S synthesizing enzyme, *cbs-1*, is required for the extended lifespan of a commonly used CR mutant (*eat-2*)^17^. To further test this link, we sought to investigate whether delivery of exogenous H_2_S by FW1256 resulted in CR-like benefits and/or tradeoffs and phenotypes in WT and *eat-2* mutants. Comparing the lifespan of unexposed and FW1256-exposed (500 µM) WT and *eat-2* mutants, we found that the lifespan of FW1256-exposed WT (mean lifespan: 23 ± 0.5 days) was nearly identical to that of unexposed *eat-2* mutants (mean lifespan: 23.7 ± 1.7 days) (Fig. 3d, Supplementary Table 2). Exogenous H_2_S delivered by donor drugs was thus capable of producing lifespan benefits in WT animals that were of the same magnitude as CR. However, strikingly, FW1256 also further extended the lifespan of already long-lived *eat-2* mutants (log rank test, *p* < 0.01; mean lifespan: 28.1 ± 1.1 days, compared to unexposed *eat-2* mutants: 23.7 ± 1.4 days, *p* < 0.001) (Fig. 3d, Supplementary Table 2). Lifespan of *eat-2* mutants was extended by 18.6%, statistically within the range of lifespan extension effects observed in WT nematodes (Fig. 1e), suggesting that lifespan benefits of exogenous H_2_S were not significantly blunted in *eat-2* mutants.

CR not only extends lifespan in many organisms but has been shown to delay and prevent ageing-associated diseases in many animals, including non-human primates^6,48^. We therefore compared effects of exposure to FW1256 in WT and *eat-2* mutants with respect to healthspan. Health status of *C. elegans* was assessed using the mobility score of Herndon et al.^49^. Observation of mobility status demonstrated that both exogenous H_2_S and CR retarded the age-dependent deterioration of mobility in *C. elegans*. Both aged WT and *eat-2* mutants exposed to 500 µM of FW1256 were healthier, on average, than aged WT control (*p* < 0.001). The differences were more pronounced as nematodes aged and were most notable between day 14 and day 28. For example, on day 22, only 26% of unexposed WT remained healthy, while 68% of FW1256-exposed WT, 69% of unexposed *eat-2* mutants and 91% of FW1256-exposed *eat-2* mutants remained healthy (Fig. 3e). While unexposed WT had an average healthspan of 18.8 days, a statistically significant longer average healthspan was observed in unexposed *eat-2* mutants (22.2 days, *p* < 0.01), FW1256-exposed WT (22.1 Days, *p* < 0.001) and FW1256-exposed *eat-2* mutants (28.3 days, *p* < 0.001) (Fig. 3f). These data suggest that the beneficial effects of FW1256 on both lifespan and healthspan are additive with the *eat-2* mutation.

### FW1256 elicits healthy longevity with fewer fitness costs than CR

**Fig. 4:**
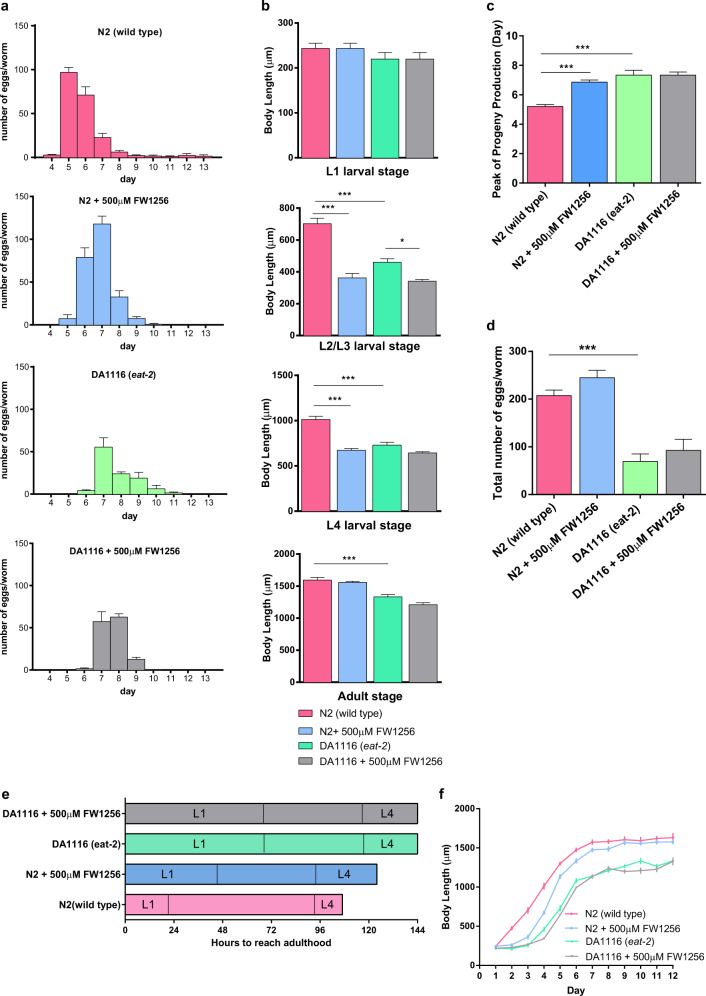
Comparing to CR, FW1256 elicits fewer fitness costs in *C. elegans*. **a** Egg laying study reveals reproduction profiles of each group of *C. elegans*. **b** Comparison of body length at L1 larval stage, L2/L3 larval stage, L4 larval stage and adult stage. **c** Day of peak progeny production. **d** Total number of eggs laid by a nematode. **e** Developmental timing and schedules from eggs into adults of WT and *eat-2* mutants (with or without exposure to 500 µM of FW1256). **f** Measurement of body length of nematodes from age day 1 (L1 larval stage) to age day 12 (adult stage). (*n* = 10 animals each, one-way ANOVA with Bonferroni’s post-test, *p < 0.05, ***p < 0.001).

In addition to beneficial and protective effects, CR is typically associated with developmental tradeoffs, in particular with reduced fecundity^50^, slower growth and smaller adult body size^51^ as well as delayed development^52^. To assess if exposure to H_2_S replicates these longevity-associated tradeoffs in *C. elegans*, we determined egg laying profiles, growth and developmental timing for WT and *eat-2* mutants with and without exposure to FW1256. Comparing the egg laying distributions (Fig. 4a), we found that both FW1256 and CR delayed reproduction by 1.7 days (Peak of progeny production: 6.9 ± 0.1 days, compared to unexposed WT: 5.2 ± 0.1 days, *p* < 0.001) and 2.1 days (Peak of progeny production: 7.3 ± 0.3 days, compared to unexposed WT: 5.2 ± 0.1 days, *p* < 0.001) respectively. However, exposure to 500 µM of FW1256 did not further delay the peak of progeny reproduction in *eat-2* mutants, compared to unexposed *eat-2* mutants (Fig. 4c). As expected, unexposed *eat-2* mutants laid significantly fewer eggs (Total number of eggs laid: 65 ± 15.7) than unexposed WT (Total number of eggs laid: 207 ± 11.6, *p* < 0.001). However, to our surprise, we found that FW1256 did not suppress total reproduction capability in either WT (Total number of eggs laid by FW1256-exposed WT: 245 ± 15.6, compared to unexposed WT: 207 ± 11.6, *p* > 0.05) or *eat-2* mutants (Total number of eggs laid by FW1256-exposed *eat-2* mutants: 93 ± 23.7, compared to unexposed *eat-2* mutants: 69 ± 15.7, *p* > 0.05) (Fig. 4d).

Exposure to FW1256 and *eat-2* mutation both resulted in developmental delay, with *eat-2* mutants being somewhat slower to develop than FW1256-exposed WT *C. elegans*, yet the exposure to FW1256 did not further delay developmental timing of *eat-2* mutants (Fig. 4e).

WT *C. elegans* grew rapidly in size between day 1 and day 6 of life, growing at a rate of 125 ± 6 µm/day, on average (Fig. 4f, Table 2). Unexposed *eat-2* mutants initially grew much more slowly and only started growing between day 3 and day 6 at a rate of 119 ± 5 µm/day. Thereafter, they stopped growing in size and remained small relative toWT for their entire life (*p* < 0.001) (Fig. 4b and f, Table 2). Exposure to FW1256 similarly initially delayed growth of WT, but between day 3 and day 6, FW1256-exposed WT animals actually grew at a faster rate compared to unexposed WT (at a rate of 142 ± 6 µm/day, *p* < 0.05). FW1256-exposed WT therefore were able to catch up in size and reached the same adult body size as unexposed WT before both stopped growing from day 9 onwards (Fig. 4b and f, Table 2).

**Table 2:** Rate of development.

Samples	Rate of development (µm/day)
N2 (wild type)	125 ± 6
N2+ 500 µM FW1256	142 ± 6
DA1116 (*eat-2*)	119 ± 5
DA1116+ 500 µM FW1256	119 ± 4

This means that, FW1256 only reduced body length of WT significantly during L2 to L4 larval stages (*p* < 0.001) whereas *eat-2* mutants had significant shorter body length than unexposed WT from L2 larval stages onwards and throughout the entire lifespan (*p* < 0.001). Finally, exposure to FW1256 did not further impact growth of *eat-2* mutants (Fig. 4b).

### FW1256 causes alterations in mitochondrial metabolism

**Fig. 5:**
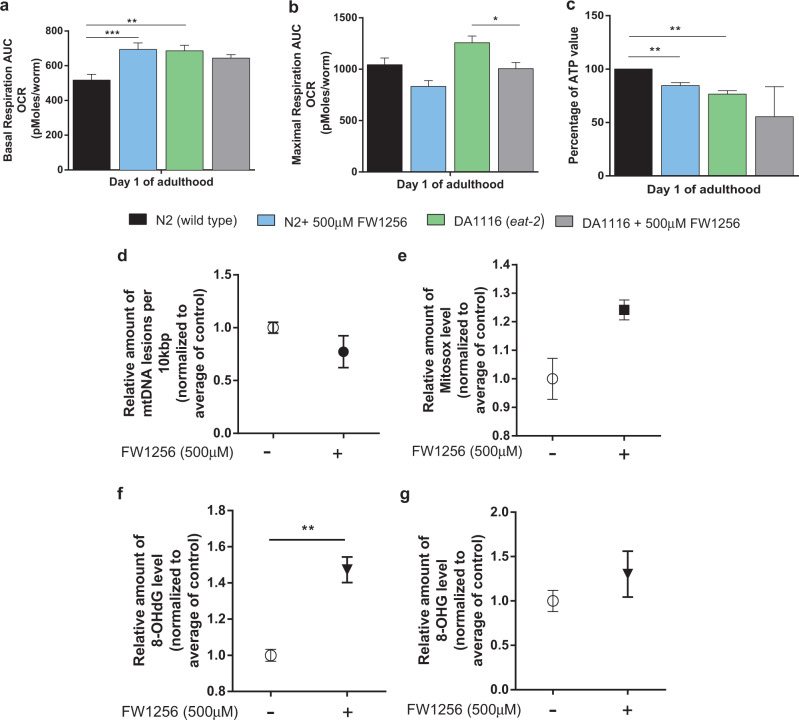
Effects of FW1256 on metabolic profiles and oxidative stress in *C. elegans*. **a–c** Comparison of metabolic parameters in WT (unexposed), *eat-2* mutants (unexposed), WT and *eat-2* exposed to FW1256 (500 µM). **a** Basal respiratory capacity (*n* = 3 biological repeats, one-way ANOVA with Bonferroni’s post-test, **p < 0.01, ***p < 0.001), **b** maximal respiratory capacity (*n* = 3 biological repeats, one-way ANOVA with Bonferroni’s post-test, *p < 0.05), **c** ATP levels (*n* = 3 biological repeats, *t*-test, **p < 0.01). **d**–**g** Measurement of oxidative stress in WT. **d** Mitochondrial DNA oxidative damage marker was measured by quantitative PCR (*n* = 3 biological repeats, mean of three separate experiments normalized to average of control, *t*-test). **e** Reactive oxygen species (ROS) production determined by mitoSOX red fluorescence (*n* = 2 biological repeats, mean of two separate experiments normalized to average of control, *t*-test). **f**, **g** general oxidative DNA damage marker, 8-OHdG (**f**) and oxidative RNA damage marker, 8-OHG (**g**) normalized to average of control (*n* = 3 biological repeats, *t*-test, **p < 0.01).

CR has previously been reported to increase mitochondrial respiration in *C. elegans*^53^, *Saccharomyces cerevisiae*^54^ and mice^55^. On the other hand, H_2_S has been reported to bind to mitochondrial Cytochrome C Oxidase, thereby inhibiting mitochondrial respiration^56^. We therefore wondered in what way exogenous H_2_S generated by FW1256 would impact respiratory capacity and ATP levels of *C. elegans*. To compare the metabolic effects of CR and FW1256 exposure, we determined both basal respiration and maximal respiration with and without exposure to FW1256 (Supplementary Fig. 8)^57^. WT animals exposed to FW1256 and *eat-2* mutants both showed significantly higher basal respiration (about 40%, *p* < 0.001; about 30%, *p* < 0.01, respectively) (Fig. 5a) compared to unexposed WT but we detected no significant change in maximal respiration rates. However, exposure to FW1256 decreased maximal respiration rate of *eat-2* mutants significantly (20%, *p* < 0.05) (Fig. 5b). To determine if significant changes in energy availability were associated with this increase in basal respiration rate, we next quantified ATP levels using the firefly luciferase assay^58^. Interestingly, we found that both WT exposed to FW1256 and *eat-2* mutants had significantly lower ATP levels compared to unexposed WT, with 16% and 24% relative reduction, respectively (*p* < 0.01) (Fig. 5c).

### Effects of FW1256 on markers of ROS and oxidative damage

The free radical theory of ageing (FRTA) proposes accumulation of free radical-mediated damage as a key driving force of the ageing process^59^. However, this view has been challenged, especially in *C. elegans*^53,60–63^. We therefore attempted to examine to what extent (if any) oxidative stress was correlated with lifespan extension by FW1256 in *C. elegans*. Four different parameters were measured to determine changes in oxidative damage and reactive oxygen species (ROS). We measured oxidative damage to mitochondrial DNA (mtDNA), using quantitative PCR (qPCR)^64^, mitochondrial superoxide *via* mitoSOX red fluorescence^33^ as well as 8-Hydroxydeoxyguanosine (8-OHdG) and 8-hydroxyguanosine (8-OHG) using liquid chromatography–mass spectrometry (LC-MS) as markers of oxidative DNA (8-OHdG) and RNA (8-OHG) damage. While we observed no significant changes in oxidative damage to mtDNA, mitoSOX fluorescence or 8-OHG (Fig. 5d, e, g), we found a significant elevation in 8-OHdG upon exposure to FW1256 (fold change 1.5, *p* < 0.01), suggesting increased DNA damage in animals exposed to FW1256 (Fig. 5f). Exposure to FW1256 therefore does not inhibit mitochondrial respiration and does not elicit an antioxidant effect but instead causes increased respiration and elevates oxidative DNA damage.

### FW1256 acts differently from CR

**Fig. 6:**
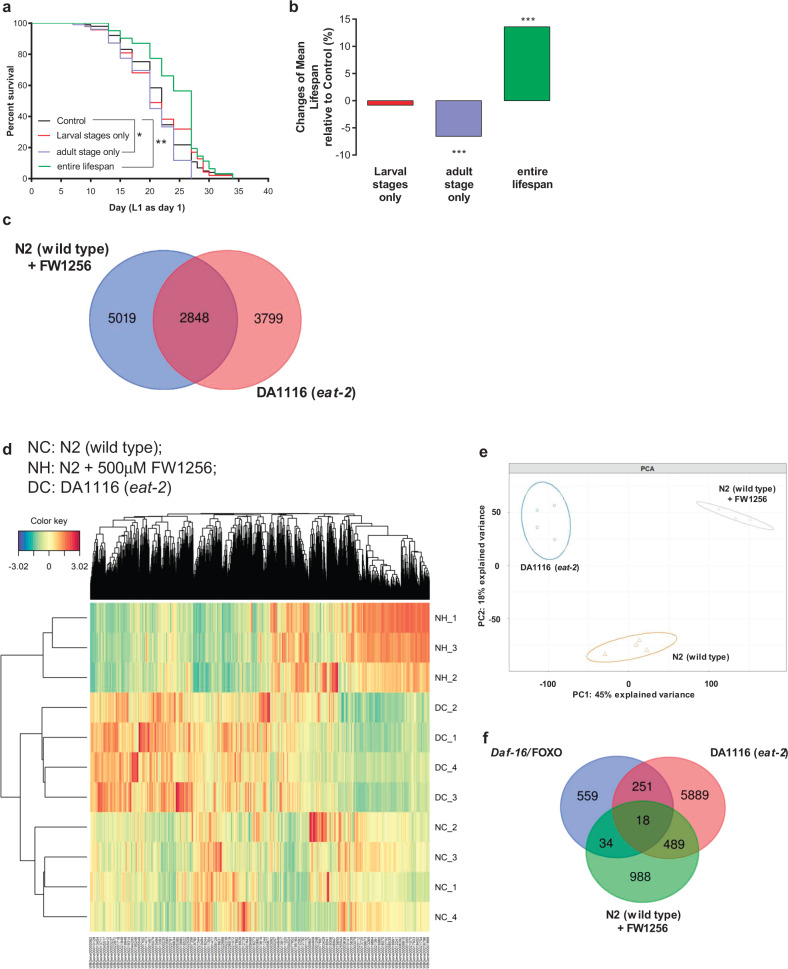
FW1256 acts differently from CR. **a** Survival curves of WT *C. elegans* upon exposure to 500 µM of FW1256 at different developmental stages: Larval only, adult stage only and entire lifespan (*n* = 47–102 animals per condition, log-rank tests, *p < 0.05, **p < 0.01). **b** Percentage change in mean lifespan relative to control (*n* = 47–102 animals per condition, mean lifespans were analyzed using OASIS2, ***p < 0.001). **c** Venn diagram of genes differentially expressed following exposure to FW1256 or in *eat-2* mutants, relative to WT controls (Hypergeometric test, ***p < 0.001). **d** Heatmap and cluster analysis of all genes with expression differences in unexposed WT, WT exposed to FW1256 and unexposed *eat-2* mutants. **e** PCA of unexposed WT, WT exposed to FW1256 and unexposed *eat-2* mutants. **f** Venn diagram of genes downstream of *Daf-16*/FOXO that are affected following exposure to FW1256 or in *eat-2* mutants (Hypergeometric test, non-significant, *p* > 0.05).

Most of the evidence above implies that the effect of exogenous H_2_S as delivered by FW1256, is not directly mimicking CR. Our data suggest a more complex interplay between endogenous H_2_S production, energy metabolism, oxidative damage, lifespan, healthspan, and developmental- and fitness- tradeoffs. Moreover, CR is typically initiated in adult animals and CR certainly has been shown to robustly benefit longevity when initiated in adults^65,66^. On the other hand, studies on H_2_S exposure in *C. elegans*, to date, have typically included exposure throughout life, starting from eggs or L1 larvae^25,33,67^. Miller and Roth have reported too that exposure to H_2_S gas, when restricted to adults alone, failed to extend lifespan of *C. elegans*^25^. We therefore wondered if, as would be expected for a true CR mimetic, exposure to FW1256 during adulthood only would still result in lifespan benefits. To systematically explore requirements for H_2_S exposure during different life stages, we compared lifespan effects following exposure to 500 µM of FW1256 during either larval or adult stages only, to benefits seen with lifelong exposure. We found that FW1256 exposure beginning at larval stage (L1) was necessary but not sufficient for longevity increase in *C. elegans* (Fig. 6a). In fact, exposure during the adult stage alone (starting after L4) was detrimental to lifespan (Fig. 6a), resulting in a significant reduction of mean lifespan (mean lifespan: 20.2 ± 0.5 days, compared to control: 21.6 ± 0.5 days, *p* < 0.001) (Fig. 6b, Supplementary Table 2) while lifelong exposure to FW1256 showed significant lifespan extension effect (mean lifespan: 24.5 ± 0.6 days, compared to control: 21.6 ± 0.5 days, *p* < 0.001) (Fig. 6b, Supplementary Table 2) and exposure during the larval stages only was neither detrimental nor beneficial (*p* > 0.05, Fig. 6a, b, Supplementary Table 2).

### FW1256 is different from CR at the transcriptional level

To further compare effects of FW1256 with those of *eat-2* mutation, we used transcriptomic analysis (RNAseq) to determine sets of differentially expressed genes (DEG) for FW1256-exposed WT and *eat-2* mutants, relative to unexposed WT animals. Comparing these DEGs showed that 2848 of 7867 genes (36%) significantly affected by FW1256 were also regulated in *eat-2* mutants, while 43% of genes in the DEG of *eat-2* mutants were also affected by exposure to FW1256 (Fig. 6c). This level of overlap is statistically significantly higher than expected by chance (*p* < 0.001) (Fig. 6c). However, since both interventions extend lifespan, it may not be surprising that they share some of the same genetic targets. A heatmap analysis further revealed that many of the gene expression changes induced by FW1256-exposure were different in direction and magnitudes from the gene expression changes induced by *eat-2* mutation (Fig. 6d). Principal component analysis (PCA) further revealed that gene expression changes following FW1256-exposure and *eat-2* mutation were well-separated, being far from each other in transcriptional space. Interestingly, in the PCA, WT exposed to FW1256 and *eat-2* mutants lie in opposite directions relative to unexposed WT, suggesting that FW1256 does not mimic global transcriptional changes of *eat-2* mutants but affects major parts of the transcriptome in a dissimilar or opposite way as CR (Fig. 6e). One of the key transcription factors thought to mediate CR benefits is *Daf-16/*FOXO^68^. Among 862 genes that were downstream of *Daf-16*/FOXO, 52 genes were regulated by FW1256 while 269 genes were regulated in *eat-2* mutants, with only 18 genes being affected by both FW1256 and *eat-2* mutation (Fig. 6f). This level of overlap is non-statistically significantly higher than expected by chance (*p* > 0.05) (Fig. 6f). Taken together, these data show that, while FW1256 affects some of the same downstream genes as *eat-2* mutation, global gene expression changes are dissimilar between FW1256-exposed animals and *eat-2* mutants, suggesting that FW1256 does not mimic CR at the transcriptional level.

## Discussion

Exposure of *C. elegans* to H_2_S gas at 50ppm extends mean lifespan by up to 70%^25^. Here show that a novel slow-releasing H_2_S donor, FW1256, which releases H_2_S more rapidly than previous compounds, extended mean lifespan by up to 51% (with an average of 33.8% across 5 experimental repeats). The observation that FW1256 is non-toxic at 500 µM is consistent with a previous report that high concentrations (up to 500 µM) of FW1256 are non-toxic in human lung fibroblasts^38^. Exposure to FW1256 also promoted health and reproduction in *C. elegans*. Although evolutionary theories imply that links between longevity benefit and fitness tradeoffs are obligatory^69,70^, our data show that FW1256 caused fewer fitness costs, at least on those aspects of fitness that we tested.

Given the evidence that H_2_S plays a role in mediating CR-associated benefits^17^, we used FW1256 to elucidate the function of H_2_S in the context of CR and to evaluate the capability of H_2_S donor compounds to replicate CR-associated effects in *C. elegans*. While FW1256 replicated some of the phenotypes of *eat-2* mutants, our data suggest that exogenous H_2_S does not completely mimic CR. We found that (i) FW1256 did not delay developmental rate substantially, (ii) FW1256 only slightly delayed reproduction but, in contrast to *eat-2* mutation, (iii) FW1256 did not reduce reproductive capacity and (iv) FW1256 did not affect the final body length of nematodes. FW1256 promoted further lifespan and healthspan extension in *eat-2* mutants and FW1256 also promoted healthy longevity with less severe fitness costs than seen in *eat-2* mutants. Furthermore, in contrast to CR, H_2_S exposure throughout the entire lifespan was required for lifespan benefits. These data imply that H_2_S is involved in regulating CR-associated effects, but that its regulatory function is more complex than appreciated previously. Finally, transcriptomics analysis showed that global transcriptional changes between WT exposed to FW1256 and *eat-2* mutants were different. This further confirmed that FW1256 and CR evoke mostly independent or only partially overlapping pathways to elicit their lifespan extension effect.

Our findings suggest that FW1256 elevated rather than reduced oxidative stress in some compartments, although only 8-OHdG was elevated significantly. The effect of FW1256 on mitochondrial ROS as evaluated by mitoSOX contrasts with previous results showing that another H_2_S releasing drug, GYY4137, reduces mitoSOX levels substantially^33^, suggesting that H_2_S does not always act as an antioxidant.

In conclusion, we have validated FW1256, a novel slow-releasing H_2_S donor, as a tool for the delivery of exogenous H_2_S. We found that delivery of exogenous H_2_S by FW1256 extended lifespan and promoted healthy ageing in *C. elegans*. These lifespan and healthspan benefits were of a similar magnitude as those typically resulting from CR. This is consistent with previous reports that H_2_S may be a key mediator of lifespan benefits associated with CR. However, the present work suggests that H_2_S releasing donors do not mimic CR completely. Since the benefits of H_2_S exposure on lifespan and healthspan are associated with fewer fitness tradeoffs, research into the role of H_2_S in longevity may reveal novel ways to modulate ageing.

## Methods

### Maintenance of *C. elegans*

The following *C. elegans* strains were used in this study: wild type (N2), RB839 (*cbs-2*), VC2569 (*cth-1*), OK2040 (*mpst-1*), DA1116 (*eat-2*). The nematodes were maintained and exposed to H_*2*_S donor compounds (FW1251 and FW1256) according to the protocol as described previously^33^. FW1251 and FW1256 were synthesized as described elsewhere^38^ and drugs were supplied by Professor Brian William Dymock and Dr. Feng Wei from the Department of Pharmacy, Faculty of Science, National University of Singapore. Briefly, synchronous cultures of nematodes were obtained by hypochlorite treatment of gravid adult nematodes as described previously^71^. Eggs obtained from hypochlorite treatment were allowed to hatch in M9 buffer with rotation at 20 rpm overnight in order to obtain synchronized larval stage 1 (L1) nematodes. Synchronized L1 stage larvae were then maintained in the liquid medium containing M9 buffer, *Escherichia coli* OP50-1, and streptomycin (200 µg/ml) in the presence or absence of H_2_S donor compounds for 48 h at 20 °C. Thereafter, nematodes were transferred and cultivated on freshly prepared standard petri-dishes containing nematode growth medium (NGM) agar seeded with *E. coli* OP50-1 (see ^58^) (For more information regarding freshly prepared NGM agar and *E. coli* OP50-1, see Supplementary Fig. 9 and Supplementary Fig. 10) with or without addition of H_2_S donor drugs.

### Lifespan determination assay

Lifespan assays were carried out as described elsewhere using randomization and operator blinding^72^. Nematodes were scored as live or dead and surviving nematodes were transferred to fresh NGM agar plates every 1 to 2 days. Death was scored based on failure to respond to gentle prodding with inoculation loop. Nematodes that died due to internal hatching, crawled off plates or lost were censored.

### Mobility status

Locomotion of 200 nematodes in each condition was assessed simultaneously as previously described^49,73^. The locomotion patterns of nematodes were classified into 3 classes. Class A animals moved constantly, class B animals only moved when prodded while class C animals showed movement of their head and tail only. Both Class A and B nematodes were scored as “healthy” whereas class C and dead nematodes were scored as unhealthy^73^. The progression from healthy to non-healthy classes was scored and the rate was utilized as the index of mobility status in each population.

### Egg laying studies

10 larvae from each condition were transferred onto individual NGM agar plates with or without FW1256 (500 µM). Thereafter, each nematode was transferred to a new NGM agar plate daily until egg laying ceased. Offspring were allowed to develop at room temperature and the number of progeny was quantified 2 days later.

### Nematode length analysis

For each condition, photographs of 10 nematodes were taken at each timepoint using a calibrated Leica MZ10F microscope. Body length of nematodes was quantified using the free curve tool, provided by the Leica Application Suite software (v2.6.0 R1).

### Developmental rates studies

The times required by about 30 to 50 eggs in each condition to become egg-laying adults (first day of adulthood) were observed.

### H_2_S detection in *C. elegans*

*Body volume*: Images of adult nematodes were taken beforehand, using a calibrated Leica MZ10F microscope. Body length and body width of nematodes were quantified using the free curve tool provided by the Leica Application Suite software (v2.6.0 R1). The body volume of nematodes was determined using the formula for the volume of a cylinder, πr^2^h.

*H*_*2*_*S detection*: Adult nematodes were incubated in M9 buffer containing 50 µM of H_2_S sensor, 7-azido-4-methylcoumarin (AzMC) (Sigma-Aldrich) at 20 °C for 2 h. AzMC fluorescence signal were visualized using confocal laser scanning microscope (Zeiss LSM800). The fluorescence intensity was then quantified using ImageJ software and normalized to body volume of nematodes.

### Quantification of ATP levels

ATP levels in 300 nematodes were measured as described previously^58^. Briefly, nematodes were collected and flash frozen. Frozen nematodes were lysed in trichloroacetic acid, followed by centrifugation at 15,000 *g* for 5 min at 4 °C. 5 μl of ATP standards or supernatants of samples were added into a white 96-well microtiter plate containing arsenite ATP buffer. Thereafter, ATP levels were quantified using a luminometer (Synergy H1, Biotek) preprogrammed to inject firefly lantern extract (2 mg/ml).

### mRNA quantification *via* real time polymerase chain reaction

Total RNA was extracted from day 1 adult nematodes using RNeasy Micro kits from Qiagen and was reverse transcribed into cDNA using oligo(dT) priming according to manufacturer’s protocol (GoScript Reverse Transcription System, Promega). Real time PCR was performed using PowerUp SYBR Green Master Mix (Life Technologies) on ViiA7 real time PCR system (Applied Biosystems). Relative fold change was determined by 2^-ΔΔCT^ method and normalized to housekeeping gene, *pmp-3*. Primer sequences as listed below were taken from ^43,74^.

*sqrd-1* Forward primer: GTGATCCTCGCAGAATTTGG

*sqrd-1* Reverse primer: GCTGGTCCATTCCAGTATCC

*ethe-1* Forward primer: TCAGTGCTCAGTTCAAAATCG

*ethe-1* Reverse primer: TGCAGATCTCAATGAATGTTCC

*pmp-3* Forward primer: TGGCCGGATGATGGTGTCGC

*pmp-3* Reverse primer: ACGAACAATGCCAAAGGCCAGC

### Mitochondrial DNA oxidative damage determination

Oxidative damage to mitochondrial DNA was determined using XL-PCR as described elsewhere^64^. Mitochondria were extracted and purified using Prepman Ultra Sample Preparation Reagent (Applied Biosystems). Thereafter, real time PCR was performed using GeneAMP XL PCR kit (applied Biosystems) to assess sequence-specific mitochondrial DNA damage, in which 6.3 kb region of the mitochondrial genome was assessed using SYBR green dye (primer sequences taken from ref. ^75^) and 71 bp region was assessed using Taqman probe

(Forward primer: GAGCGTCATTTATTGGGAAGAAGA

Reverse primer: TGTGCTAATCCCATAAATGTAACCTT).

### ROS quantification

ROS production in *C. elegans* was measured as described^33^ by using MitoSOX Red mitochondrial superoxide indicator (Life Technologies). 100 nematodes were transferred manually into each well of a black 96-well microtiter plate containing 100 µl of M9 buffer and 100 µl of 20 µM MitoSOX red reagent. Thereafter, ROS-associated fluorescence levels were measured every 2 min for 5 h using a fluorescence plate reader (Synergy H1 multimode microplate reader, Biotek) at excitation 396 nm and emission 579 nm at room temperature.

### Respiratory capacity determination

Respiratory capacity in live nematodes was determined as described elsewhere^57^. Briefly, 10 nematodes were transferred into each well of a XF96 microplate containing 200 µl of M9 buffer. Thereafter, oxygen consumption rates (OCR) were measured using XF96 extracellular flux analyzer (Seahorse Bioscience) according to manufacturer’s instruction with the injection of 25 µl of 90 µM carbonyl cyanide 4-(trifluoromethoxy) phenylhydrazone (FCCP) (Sigma-Aldrich) and 25 µl of 500 mM sodium azide (Sigma-Aldrich) sequentially. Basal respiration and maximal respiration were determined *via* quantifying area under curve of OCR with and without addition of FCCP.

### 8-OHdG and 8-OHG measurement

Frozen nematodes were sonicated in lysis buffer and DNA was extracted with phenol-chloroform. 100 µg of DNA (Tris pH8) and heavy labelled internal standards were mixed and hydrolyzed according to^76^. Samples were deproteinized with methanol and evaporated under nitrogen. Hydrolyzed nucleosides were dissolved in water for analysis using an Agilent 1200 System coupled to 6460 ESI tandem MS. 10 µl of the samples were injected into Accucore C18 (150 × 3.0 mm, Thermo Scientific) at 30 °C. Solvent A was Acetonitrile and Solvent B was 0.1% Formic Acid. Chromatographic separation was carried out at 0.5 ml/min using the following gradient elution: 1.5 minutes of 98% B followed by 4 min gradient decrease to 95% B, wash with 5% A for 2.5 min before 98% B for equilibration. Total run time was 12 minutes, with 8-OHG, dG, and 8-OHdG eluted at 3.6, 3.3 and 4.7 min, respectively. Mass spectrometry was carried out under positive ion ESI and multiple reaction monitoring (MRM) mode, at 3000 V and 50 psi, 350 °C, 12 L/min nitrogen nebulizer. Ultra-high purity nitrogen was used as collision gas. The compound product ion transitions are listed in Supplementary Table 3.

### RNA sequencing

Approximately 1000 of adult nematodes were harvested and total RNA was extracted using RNeasy Micro kits from Qiagen. Extracted RNA was thereafter sent to NovogeneAIT Genomics Singapore for library prep and sequencing using Illumina HiSeq4000 sequencing platform (Illumina) in a paired end read approach at a read length of 150 nucleotides. The RNAseq reads from each sample were mapped to the reference *C. elegans* transcriptome (WBcel235) with *kallisto* (v0.46.0)^77^. The estimated counts were imported from kallisto to the R environment (v3.6) and summarized to gene-level in length scaled TPM units using the *tximport* package (v1.12.3)^78^. The *DESeq2* package (v1.24.0)^79^ was used to identify differentially expressed genes (DEGs) while the *mixOmics* package (6.8.0)^80^ was used to obtain PCA plot and heatmap.

### Statistical analysis

All data were analyzed using GraphPad Prism version 5.02 software except for mean lifespan data. Lifespan curves were plotted using Kaplan–Meier survival curves and analyzed using log-rank tests while mean lifespans were plotted using GraphPad Prism version 5.02 software and analyzed using OASIS 2 (Online Application for Survival Analysis 2; https://sbi.postech.ac.kr/oasis2)^81^. All other data are presented and plotted as mean ± SEM of at least three separate experiments, analyzed using one-way ANOVA and Bonferroni’s multiple comparisons post-test, unless otherwise noted. Statistical differences with *p* < 0.05 were considered significant.

### Reporting Summary

Further information on research design is available in the Nature Research Reporting Summary linked to this article.

## Supplementary information

Supplementary Information

reporting summary

## Data Availability

The data that support the findings of this study are available from the corresponding author upon request. All RNA-Seq data were deposited in the National Center for Biotechnology Information Gene Expression Omnibus (GEO) (accession number GSE146412).
