# Supplementary material for: Lifespan and healthspan benefits of exogenous H2S in C. elegans are independent from effects downstream of eat-2 mutation
Source: NPJ Aging Mech Dis. 2020 Jun 10;6:6. doi: 10.1038/s41514-020-0044-8 (PMC7287109; doi:10.1038/s41514-020-0044-8)
Supplement: Supplementary file 1 — Supplementary Information [file 41514_2020_44_MOESM1_ESM.pdf]

Supplementary Information

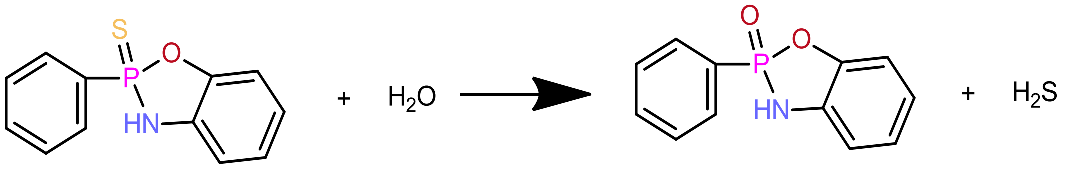

Supplementary Figure 1. Mechanism for H_2S release by FW1256 in aqueous environment.

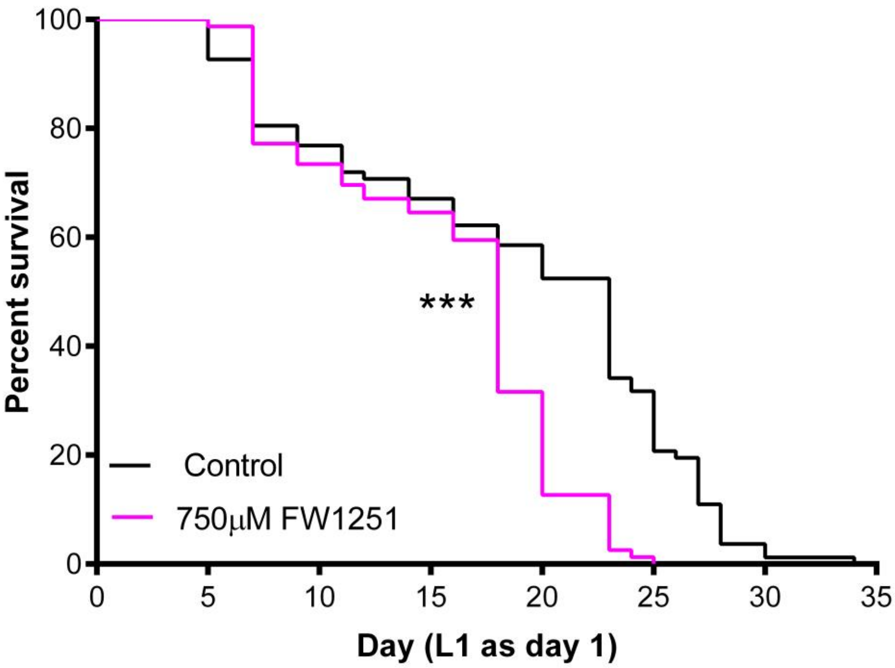

Supplementary Figure 2. Toxic effect of 750μM FW1251 on lifespan of WT *C. elegans* (Survival curves were analysed using log-rank tests, *** $p<0.001$).

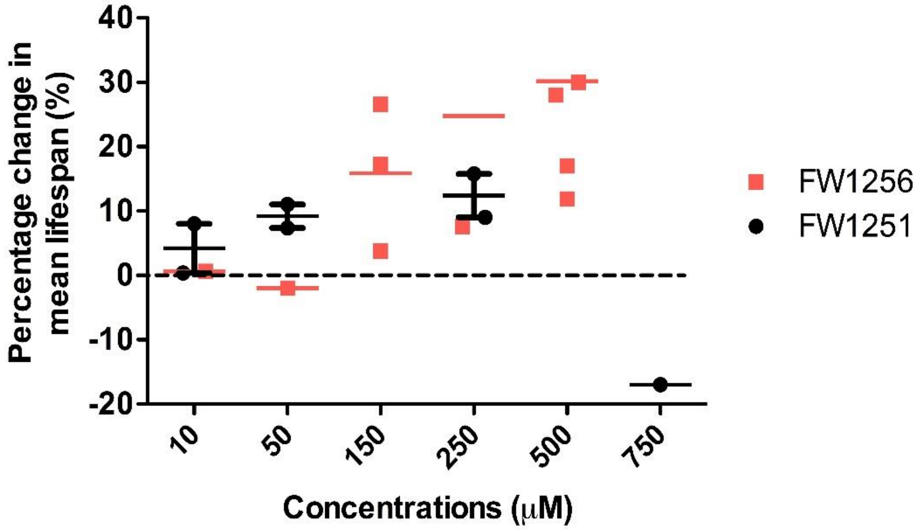

Supplementary Figure 3. Percentage change in mean lifespan of *C. elegans* upon exposure to FW1251 (10µM, 50µM, 250µM, 750µM) or FW1256 (10µM, 50µM, 150µM, 250µM, 500µM) across all conditions tested.

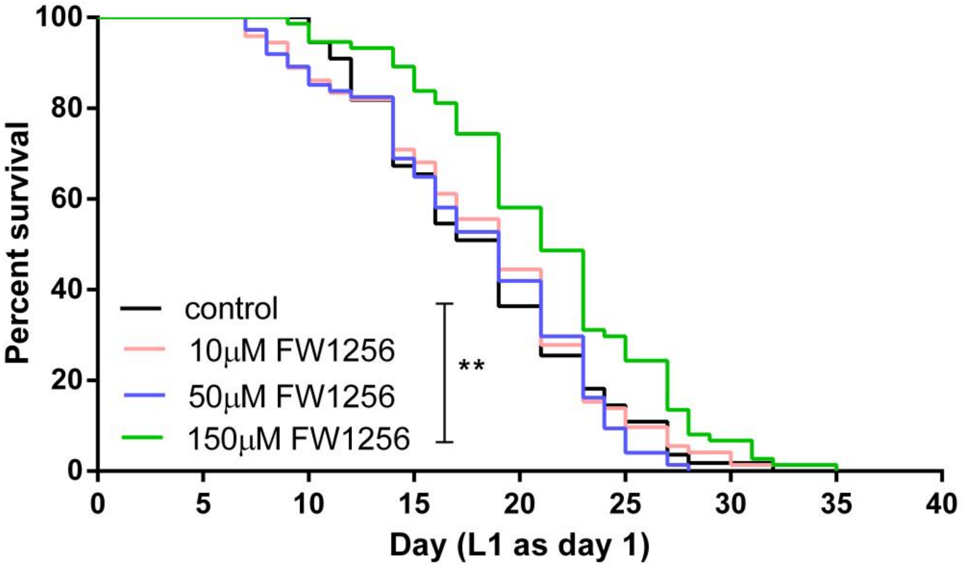

Supplementary Figure 4. Effects of FW1256 on lifespan of WT *C. elegans* at concentrations of 150μM and below (Survival curves were analysed using log-rank tests, ** p<0.01) (See Supplementary Table 2).

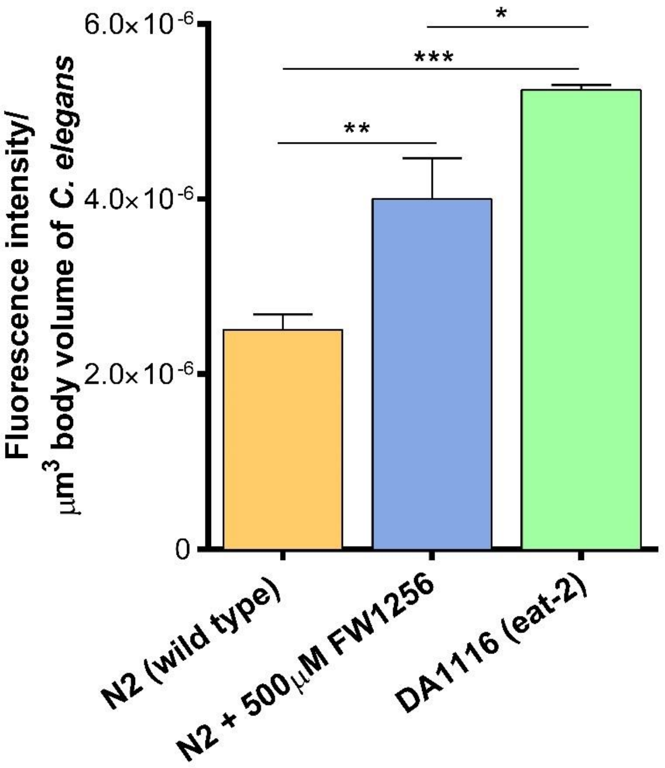

Supplementary Figure 5. Fluorescence intensity of the AzMC signal, normalized to body volume of *C. elegans*. Amount of increase in H_2S-related fluorescence seen in *eat-2* mutants is comparable to the FW1256-induced increase as judged by AzMC in wild-type nematodes exposed to 500µM of FW1256. (One-way ANOVA with Bonferroni's post-test, * p<0.05, **p<0.01, *** p<0.001).

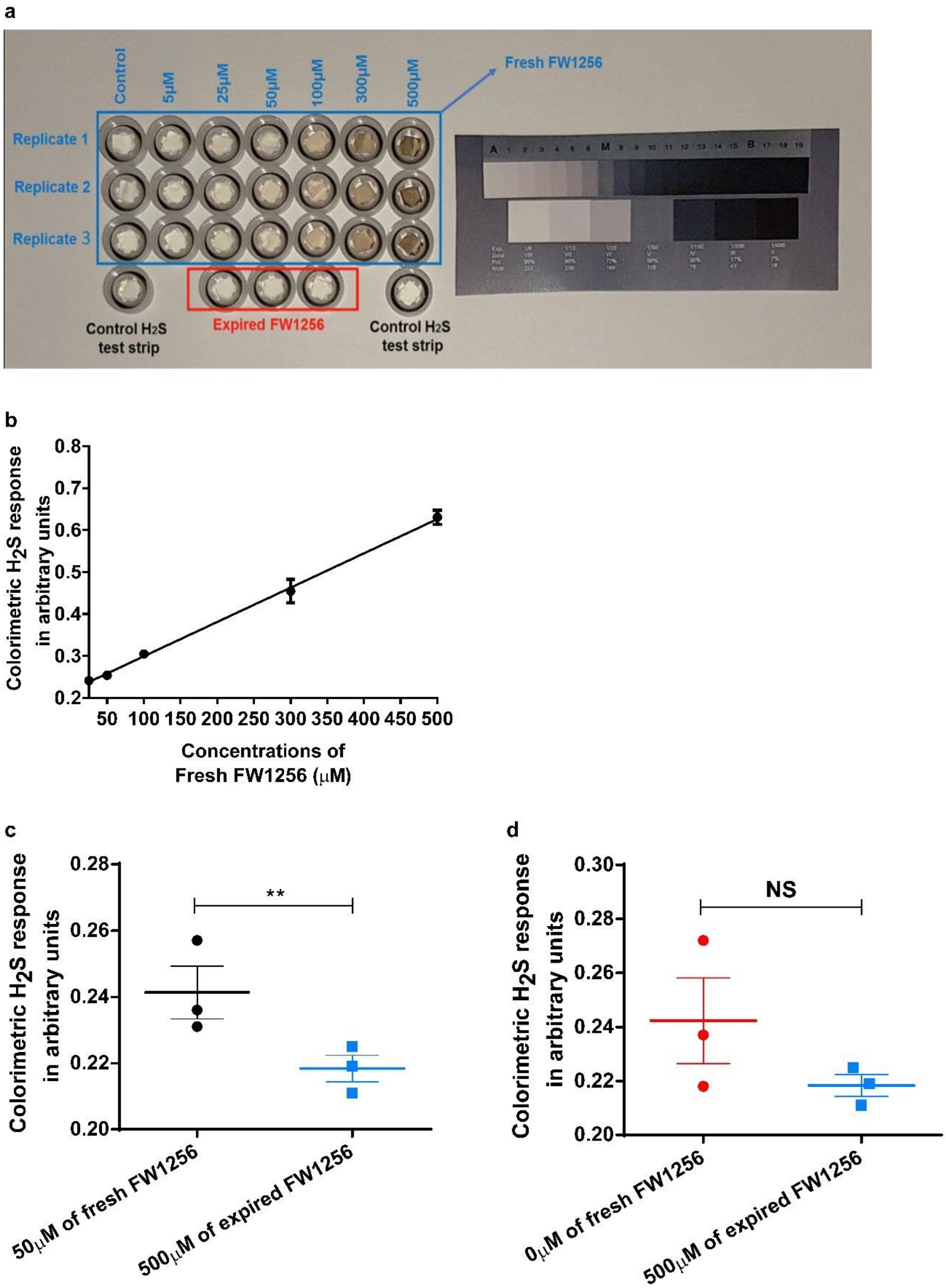
a
Control
5µM
25µM
50µM
100µM
300µM
500µM
Fresh FW1256
Replicate 1
Replicate 2
Replicate 3
Control H2S test strip
Expired FW1256
Control H2S test strip
b
Colorimetric H2S response in arbitrary units
Concentrations of Fresh FW1256 (µM)
c
Colorimetric H2S response in arbitrary units
**
50µM of fresh FW1256
500µM of expired FW1256
d
Colorimetric H2S response in arbitrary units
NS
0µM of fresh FW1256
500µM of expired FW1256

Supplementary Figure 6. H_2S detection from fresh FW1256 and expired FW1256 using H_2S test strips. **a.** Comparison of H_2S release from fresh FW1256 and expired FW1256 using H_2S test strips. Overview showing colour changes observed in H_2S test strips caused by exposure to H_2S released by different concentrations of fresh FW1256, compared to colour response seen for expired FW1256. **b.** Standard curve generated based on quantification using ImageJ of H_2S test strips from a dilution series from fresh FW1256 stock. Test strips were exposed overnight to H_2S released by a dilution series of fresh FW1256 samples **c.** Comparison between 50µM of fresh FW1256 and 500µM of expired FW1256 (t-test, ** $p<0.01$). 500µM of expired FW1256 released significantly less H_2S than 50µM of fresh stock, suggesting that H_2S released from expired FW1256 was more than 90% diminished. **d.** Comparison between 0µM of fresh FW1256 and 500µM of expired FW1256 (t-test, NS $p>0.05$). H_2S released from 500µM of expired FW1256 was statistically insignificantly different from 0µM fresh FW1256 stock.

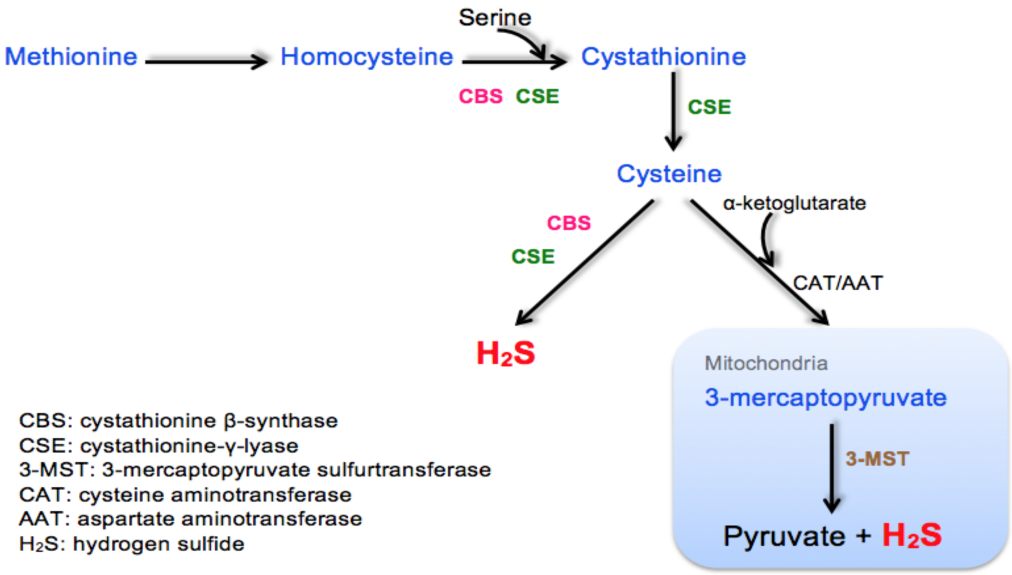

Supplementary Figure 7. Schematic diagram of endogenous H_2S synthesis system.

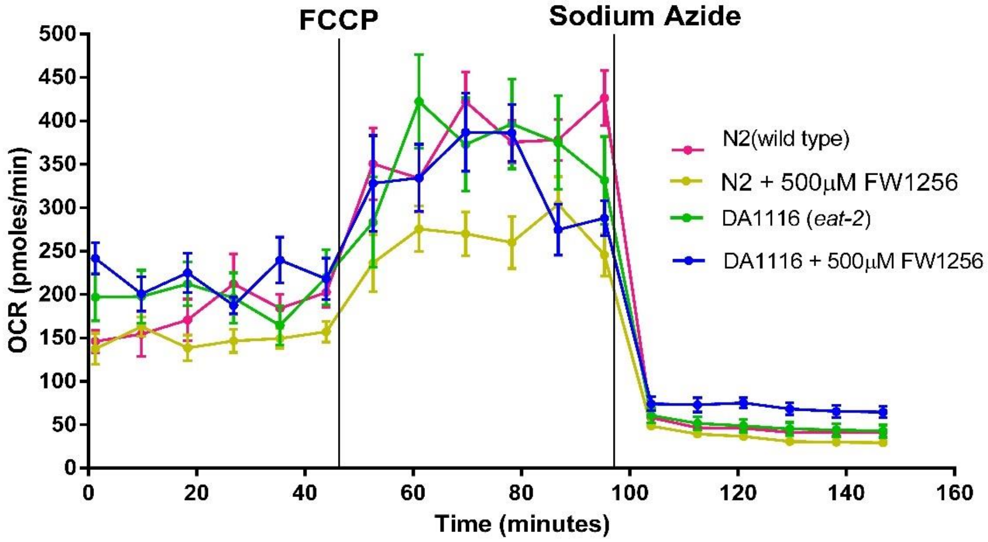

Supplementary Figure 8. Example curve from a seahorse experiment to determine basal and maximal respiratory capacity in *C. elegans*.

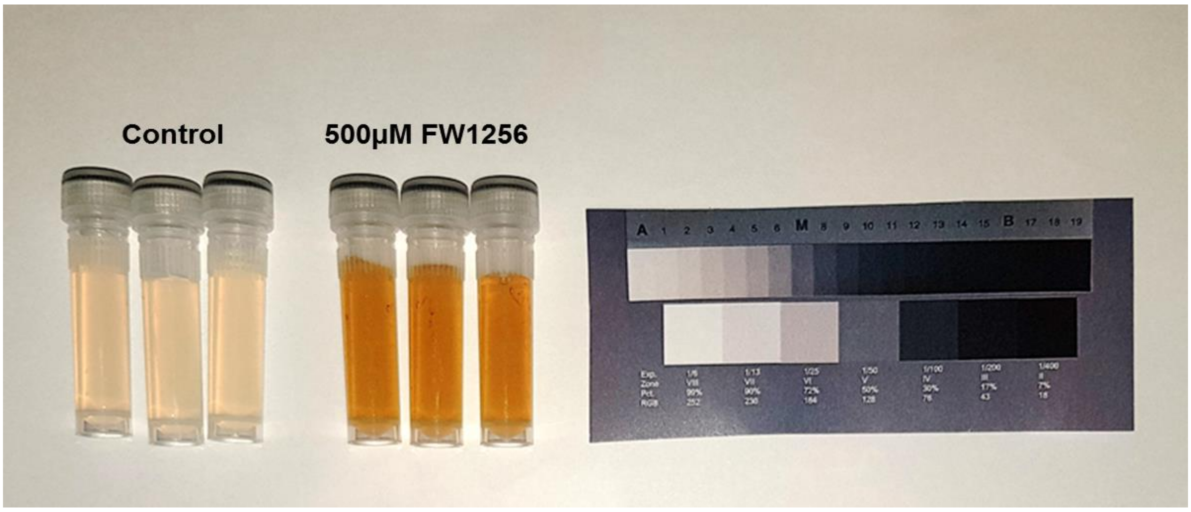

Supplementary Figure 9. Change of colour observed in NGM agar after storing at 4°C for a month. The colour of NGM agar without FW1256 remained unchanged whereas NGM agar with 500µM of FW1256 changed to brown after storing it at 4°C for a month. This change occurred gradual over time and was associated with a noticeable decrease in the intensity of the odour associated with release of H_2S. We therefore concluded that making fresh NGM agar plates weekly is essential to ensure that the amount of H_2S released is consistent over extended period of time.

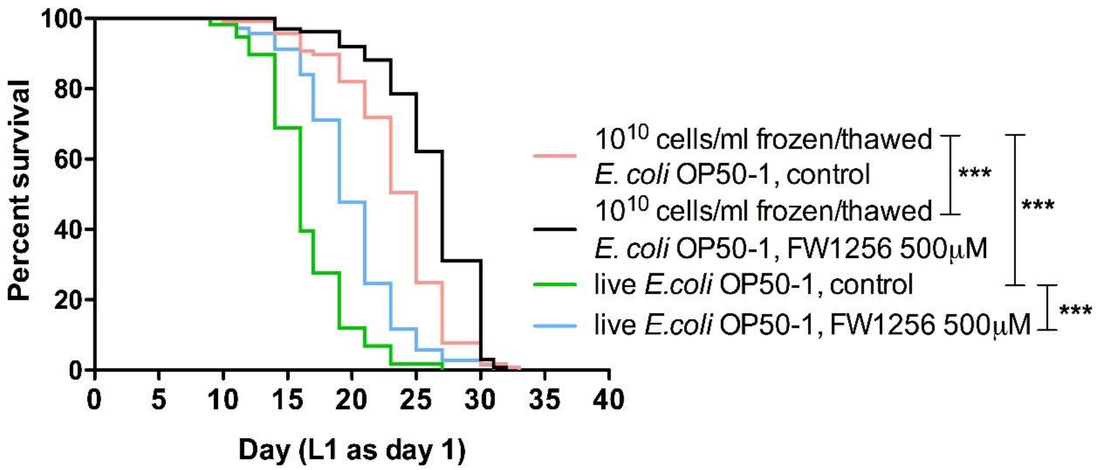

Supplementary Figure 10. Effects of FW1256 exposure on lifespan of WT grown on 10^{10}cells/ml of frozen/thawed *E. coli* OP50-1 and live *E. coli* OP50-1 (grown overnight *in situ* on NGM agar plates[71]. While baseline lifespans on these food-sources are different, the types of *E. coli* OP50-1 did not affect the relative efficacy of FW1256 in terms of lifespan-extension (Survival curves were analysed using log-rank tests, *** $p<0.001$) (See Supplementary Table 2).

Supplementary Table 1. Summary of lifespan effects in five independent repeats of exposure to FW1256 (500µM).

Trials	Mean Lifespan ± SEM (days)		Percentage extension (%)	Log-rank test
	Control	500µM FW1256		
**1**	16.9 ± 0.4, n=111	25.6 ± 0.3, n=150	51	<0.001
**2**	16.8 ± 0.3, n=137	24.1 ± 0.2, n=175	43	<0.001
**3**	17.9 ± 0.3, n=147	23.3 ± 0.4, n=133	30	<0.001
**4**	18.5 ± 0.3, n=118	23.7 ± 0.3, n=110	28	<0.001
**5**	19.8 ± 0.3, n=122	23.1 ± 0.5, n=94	17	<0.001

Supplementary Table 2. Summary of lifespan studies

Figure	Strains	Condition	n	Mean Lifespan ± SEM (days), *p*-value compared to N2 control	Log-rank test, *p*-value compared to N2 control
**1a**	N2	Control	137	17.9 ± 0.5	
		10μM FW1251	145	19.4 ± 0.5, <0.05	<0.05
		50μM FW1251	117	19.9 ± 0.5, <0.05	<0.05
		250μM FW1251	103	19.5 ± 0.7, <0.01	<0.001
**1c**	N2	Control	111	16.9 ± 0.4	
		150μM FW1256	137	21.4 ± 0.3, <0.001	<0.001
		250μM FW1256	141	24.0 ± 0.3, <0.001	<0.001
		500μM FW1256	150	25.6 ± 0.3, <0.001	<0.001
**2d**	N2	Control	163	19.9 ± 0.3	
		500μM FW1256	127	22.9 ± 0.4, <0.001	<0.001
		500μM FW1256 (time-expired)	102	17.1 ± 0.4, <0.001	<0.001

**3a-c**	N2	Control	147	17.9 ± 0.3	
	RB839	Control	115	17.2 ± 0.3, >0.05	>0.05
		500µM FW1256	125	26.9 ± 0.5, <0.001[a]	<0.001[a]
	VC2569	Control	142	18.2 ± 0.3, >0.05	>0.05
		500µM FW1256	132	24.3 ± 0.4, <0.001[b]	<0.001[b]
	OK2040	Control	170	15.4 ± 0.2, <0.001	<0.001
		500µM FW1256	181	22.4 ± 0.3, <0.001[c]	<0.001[c]
**3d**	N2	Control	122	19.8 ± 0.3	
		500µM FW1256	94	23.1 ± 0.5, <0.001	<0.001
	DA1116	Control	27	23.7 ± 1.4, <0.001	<0.001
		500µM FW1256	63	28.1 ± 1.1, <0.001[d]	<0.01[d]
**6a**	N2	Control	101	21.6 ± 0.5	
		L4 only	47	21.4 ± 0.9, >0.05	>0.05
		Adult only	102	20.2 ± 0.5, <0.001	<0.05
		Entire lifespan	62	24.5 ± 0.6, <0.001	<0.01

**Supplementary Figure 2**	N2	Control	82	18.8 ± 0.9	
		750μM FW1256	79	15.6 ± 0.7, <0.001	<0.001
**Supplementary Figure 4**	N2	Control	55	18.3 ± 0.7	
		10μM FW1256	72	18.4 ± 0.7, >0.05	>0.05
		50μM FW1256	74	17.9 ± 0.6, >0.05	>0.05
		150μM FW1256	74	21.4 ± 0.7, <0.01	<0.01
**Supplementary Figure 10**	N2	Frozen/thawed *E.coli* OP50-1, Control	122	23.4 ± 0.4	
		Frozen/thawed *E.coli* OP50-1, 500μM FW1256	94	26.2 ± 0.3, <0.001	<0.001
	N2	Live *E. coli* OP50-1, Control	27	16.5 ± 0.4	
		Live *E. coli* OP50-1, 500μM FW1256	63	19.9 ± 0.5, <0.001	<0.001

[a]compared to control RB839. [b]compared to control VC2569. [c]compared to control OK2040. [d]compared to control DA1116.

n represents number of animals tested.

Supplementary Table 3. Product ion transitions for each compound

	**MRM transition**	**Dwell Time (ms)**	**Fragmentor (V)**	**Collision Energy (eV)**
**8OHG-ISTD**	303 → 171	60	85	10
**8OHG**	300 → 168	60	85	10
**8OHdG-ISTD**	287 → 171	60	80	10
**8OHdG**	284 → 168	60	80	10
**dG-ISTD**	271 → 155	60	65	5
**dG**	268 → 152	60	70	6
